# *Propionibacterium freudenreichii* MJ2 Improves Dexamethasone-Induced Muscle Atrophy in Rats by Increasing Muscle Mass and Muscle Fiber Area

**DOI:** 10.4014/jmb.2510.10050

**Published:** 2026-01-18

**Authors:** Sang-Hun Kim, Hee-Eun Woo, Mirae An, Young- Hee Lim

**Affiliations:** 1 Department of Integrated Biomedical and Life Sciences, Graduate School, Korea University, Seoul 02841, Republic of Korea; 2 Department of Public Health Science, Graduate School, Korea University, Seoul 02841, Republic of Korea

**Keywords:** *Propionibacterium freudenreichii* MJ2, Muscle atrophy, Dexamethasone, Myotube, Muscle mass

## Abstract

Muscle atrophy refers to the loss or wasting of muscle tissue caused by medication, aging, disease, and injury. *Propionibacterium freudenreichii* MJ2 (MJ2) shows anti-inflammatory and anti-obesity properties. This study aimed to determine the effects of MJ2 on dexamethasone (DEX)-induced muscle atrophy in C2C12 myoblast cell line and rats. Heat-killed *P. freudenreichii* MJ2 (HK-MJ2) inhibited a decrease in the diameter of DEX-treated C2C12 myotubes. Additionally, it downregulated the expression of muscle atrophy- and apoptosis-related genes (*MuRF-1*, *Atrogin-1*, and *Bax/Bcl-2*) in DEX-treated C2C12 cells while activating proteins (p-Akt/Akt and p-mTOR/mTOR) associated with muscle protein synthesis. Live- and HK-MJ2 treatment increased grip strength, muscle mass, and muscle fiber area in rats with DEX-induced muscle atrophy. In conclusion, both live- and HK-MJ2 increase muscle mass and muscle fiber area, resulting in the improvement of DEX-induced muscle atrophy.

## Introduction

Muscle atrophy refers to the thinning and wasting of muscle tissues [1]. It is initiated by a decrease in muscle tension, followed by reduced muscle protein synthesis and increased muscle protein degradation. Various proteolytic pathways, such as the caspase system, autophagy, and ubiquitin-proteasome-dependent pathways, have been implicated in muscle atrophy. Especially, the ubiquitin-proteasome system (UPS), which drives the degradation of muscle proteins, is the most influential mechanism in muscle atrophy [2]. Muscle degradation factors F-box (MAFbx/Atrogin-1) and muscle RING-finger protein-1 (MuRF-1) are involved in the ubiquitination process and promote muscle atrophy [3].

To develop muscle atrophy animal models, several disease-inducing methods, such as starvation, casting, denervation, diabetes, and glucocorticoid administration, have been used [4]. Among these, a high concentration of dexamethasone (DEX, a representative glucocorticoid) is mainly used as a stimulus for muscle protein decomposition, which alters mitochondrial function in skeletal muscle, resulting in muscle atrophy [5]. DEX-induced protein degradation is primarily caused by the activation of lysosomal pathways and the ubiquitin-proteasome system. Specifically, DEX stimulates factors that promote muscle loss. In contrast, DEX is used to treat several diseases, such as certain forms of arthritis, intestinal disorders, allergies, and asthma. However, long-term use of high-dose DEX may increase the expression of muscle-specific E3 ubiquitin ligases, MuRF-1, and MAFbx/Atrogin-1, ultimately leading to muscle atrophy [6]. Therefore, muscle atrophy induced by high-dose DEX has been used to screen for agents capable of improving muscle atrophy. Additionally, DEX treatment serves as a useful method for developing cellular or animal models to study the pathogenesis of muscle atrophy.

Recently, there has been an increase in the number of elderly people and a reduction in outdoor activities owing to the impact of pandemic infectious diseases, leading to an increase in the incidence of muscle atrophy worldwide. The occurrence of muscle atrophy is influenced by various factors, including genetics, various diseases, long-term inactivity, cancer, and aging [7]. Muscle atrophy can significantly decrease the quality of life and functional status of the body and increase the rate of mortality. There are several links between obesity and muscle atrophy. Osteoporosis is associated with muscle fiber atrophy [8] and bone loss because of various causes, such as limited musculoskeletal performance, exposure to microgravity, and aging, which is preceded by a rapid loss of muscle mass [9]. Despite many studies being conducted to prevent and treat muscle atrophy, the prevention and treatment of muscle atrophy remain unresolved. The use of current therapeutics for treating musculoskeletal diseases is limited owing to their limited treatment effects and potential side effects. Therefore, there is a demand for the development of safe and effective treatments to prevent the loss of skeletal muscle, which leads to muscle atrophy. While probiotics are utilized as therapeutic agents for various diseases, the development of microbial materials for the treatment of musculoskeletal diseases remains understudied. However, there have been reports that supplementation of probiotics shows enhancement of muscle mass and muscle strength [10, 11]. Recently, live and inactivated probiotics show similar health benefits [12-14]. Inactivated probiotics (called as paraprobiotics) obviously have some merits over live probiotics, for instance, stability, no infection in compromised individuals, stability, and ease of production, transportation, and storage, while still retaining health-promoting properties comparable to their viable counterparts. Currently, inactivated probiotics are receiving attention for their health promotion effects, but the available information is limited.

*Propionibacterium freudenreichii* is a gram-positive probiotic bacterium that is classified as Generally Recognized As Safe (GRAS). It is utilized in the production of fermented dairy products and commonly serves as a ripening starter for hard-type cheeses. *P. freudenreichii* produces bifidogenic compounds, such as propionic acid and vitamin B12, that affect immune regulation. This characteristic has drawn attention to *P. freudenreichii* as a potential next-generation probiotic. *P. freudenreichii* MJ2 (MJ2), isolated from raw milk, has shown anti-obesity effects [15] and bone mineralization enhancement [16]. Therefore, in this study, we evaluated whether MJ2 prevents or improves muscle atrophy using the myoblast cell line C2C12 and rats as *in vitro* and *in vivo* models of DEX-induced muscle atrophy, respectively.

## Materials and Methods

### Preparation of Heat-Killed *P. freudenreichii* MJ2 (HK-MJ2)

*P. freudenreichii* MJ2 (KCCM12272P) (MJ2) was obtained from the Korean Culture Center of Microorganisms (KCCM, Republic of Korea) [16] and cultured in reinforced Clostridium medium (RCM) (Oxoid, UK) within an anaerobic conditioned chamber (GasPak EZ container system) (BD, USA) at 30°C for 48 h. Following incubation, MJ2 cells were collected by centrifugation at 4,000 ×g at 4°C for 5 min and then washed twice with phosphate buffered saline (PBS). The cells acquired in PBS were heated at 100°C for 30 min to generate HK-MJ2. Subsequently, we confirmed that there was no growth of HK-MJ2.

### C2C12 Cell Culture and Differentiation into Myotubes

C2C12 myoblast cells (ATCC, USA) were cultured in Dulbecco’s modified Eagle’s medium (DMEM) supplemented with 10% (v/v) fetal bovine serum (FBS), 100 μg/ml streptomycin, and 100 units/ml penicillin (HyClone, USA) at 37°C in a humidified 5% CO_2_ atmosphere. To induce differentiation, C2C12 cells were cultured until they reached 95% confluence. Subsequently, differentiation was induced by replacing the culture medium with the differentiation medium (DMEM with 2% horse serum (HS), 100 μg/ml streptomycin, and 100 units/ml penicillin). Throughout the differentiation period, the medium was replaced with fresh differentiation medium every 2 days until day 6.

### Cell Viability Test

Following differentiation, the culture medium was changed with serum-free DMEM containing various concentrations of HK-MJ2 (10^6^, 10^7^, and 10^8^ cells/ml) and 100 μM DEX (Sigma-Aldrich, USA). Subsequently, C2C12 cells were cultured at 37°C under 5% CO_2_ atmosphere for 24 h. After discarding the medium, cells were treated with 3-[4,5-dimethylthiazol-2-yl]-2,5-diphenyltetrazolium bromide (MTT) (125 μg/ml) (Amresco, USA) dissolved in DMEM and cultured for 2 h under the same conditions. Cell viability was determined using the manufacturer’s protocol and calculated as the percentage relative to the negative control.

### Myotube Diameter

C2C12 cells were initially seeded at a concentration of 2 × 10^5^ cells/ml in 6-well plates and cultured for 24–48 h in DMEM supplemented with 10% FBS, 100 μg/ml streptomycin, and 100 units/ml penicillin until they reached 95% confluence. Differentiation was induced by replacing 10% FBS in the culture medium with 2% HS, and this process was completed over a period of 6 days. Thereafter, the cells were treated with DEX alone (100 μM) or DEX (100 μM) in combination with HK-MJ2 (10^6^, 10^7^, and 10^8^ cells/ml). After culturing for 24 h, the C2C12 myotubes were washed with PBS, and their images were obtained using a microscope. To ensure statistical reliability, images were captured from at least three randomly selected fields per well. The diameters of randomly selected 50 myotubes per condition were measured using ImageJ software (NIH, USA).

### Quantitative Real-Time Polymerase Chain Reaction (qPCR)

Total RNA was extracted from myotubes using the RiboEx reagent (GeneAll Biotechnology, Republic of Korea), according to the manufacturer’s instructions. For cDNA synthesis, the RevertAid First Strand cDNA Synthesis Kit (Thermo Fisher Scientific, USA) was used. Subsequently, a qPCR was performed using the Kapa SYBR Fast qPCR Kit (Kapa Biosystems, USA) and QuantStudio 6 Flex System (Thermo Fisher Scientific). The reaction mixture was initially preheated at 95°C for 10 min, followed by 40 cycles at 95°C for 15 sec, 60°C for 15 sec, and 72°C for 30 sec. The primer sequences used in this study are as follows: *GAPDH*, forward 5′-GTCATCATCTCCGCCCCTTCTGC and reverse 5′-GATGCCTGC TCACCACCTTCTTG; *MuRF-1*, forward 5′-ACGAGAAGAAGAGCGAGCTG and reverse 5′-CTTGGCACTTGAGAGGAAGG; *Atrogin-1*, forward 5′-AGAGTCGGCAAGTCTGTGCT and reverse 5′-TGTAAGCACACAGGCAGGTC; *Bax*, forward 5′-TGCAGAGGATGATTGCTGAC and reverse 5′-GATCAGCTCGGGCACTTTAG; *Bcl2*, forward 5′-GGTGGTGGAGGAACTCTTCA and reverse 5′-ATGCCGGTTCAGGTA CTCAG. Relative gene expression was quantified using equal amounts of RNA, with glyceraldehyde-3-phosphate dehydrogenase (*GAPDH*) used as the reference gene. The normalized expression change was calculated as 2^-ΔΔCt^ (GAPDH control set to 1) [17].

### Western Blot Analysis

Total protein was extracted from the myotubes using the PRO-PREP Protein Extraction Solution (iNtRON, Republic of Korea), along with Halt protease inhibitor single-use cocktail (Thermo Fisher Scientific). Following centrifugation, proteins in the supernatant were quantified using the Bradford Protein Assay (Bio-Rad, USA). For electrophoresis, an equal amount (40 μg) of each protein sample was loaded onto a 10% sodium dodecyl sulfate-polyacrylamide (SDS-PAGE) gel. The separated proteins were subsequently transferred onto a polyvinylidene difluoride (PVDF membrane; Sigma-Aldrich), which was then blocked with 5% skim milk (NEOGEN Corporation, USA) in Tris-buffered saline with 0.05% Tween 20 (TBST) for at least 1 h. Subsequently, the membrane were washed thrice with TBST and incubated overnight at 4°C with primary antibodies against GAPDH (1:5000 dilution, GTX 100118, GeneTex, USA), p-Akt (protein kinase B) (Ser 473) (1:1000 dilution, sc-514032, Santa Cruz Biotechnology, USA), Akt (1:1000 dilution, MA5-14898, Invitrogen, USA), p-mTOR (mammalian target of rapamycin) (Ser 2448) (1:1000 dilution, 44-1125G, Invitrogen), and mTOR (1:1000 dilution, PA5-34663, Invitrogen). After washing the membrane thrice with TBST, it was incubated with the HRP-conjugated goat anti-rabbit IgG secondary antibody (1:2000 dilution, GTX213110-01, GeneTex) at room temperature for 1 h. After washing thrice with TBST, the bands were detected using the SuperSignal West Femto Maximum Sensitivity Substrate (Thermo Fisher Scientific). The images were captured using the FluorChem E System (ProteinSimple, USA), and band densities were measured using the ImageJ software (NIH).

### Experimental Animals

Eight-week-old male Sprague Dawley (SD) rats were purchased from Central Lab Animal, Inc. (Republic of Korea). The rats were housed under conditions of 22 ± 1°C temperature, 50 ± 5% relative humidity, and 200 ± 50 Lx illumination intensity, with a 12-h day and night cycle. Throughout the study, all rats had ad libitum access to water and food. All experimental procedures were approved by the Korea University Institutional Animal Care and Use Committee (approval number: KUIACUC-2021-0086) and were performed in accordance with the Guide for the Care and Use of Laboratory Animals (NIH Publication No. 85-23, 1996). The rats were randomly divided into seven groups (*n* = 10 per group). Group 1: normal control (NC), rats administered only vehicle (phosphate buffered saline, PBS); Group 2: DEX-treated group (DEX), rats intraperitoneally administered DEX (2 mg/kg); Group 3: test control (LEU), rats administered leucine (600 mg/kg) orally and DEX (2 mg/kg) intraperitoneally; Group 4: low-dose HK-MJ2 (LDDMJ), rats administered HK-MJ2 (10^7^ cells/ml) orally and DEX (2 mg/kg) intraperitoneally; Group 5: high-dose HK-MJ2 (HDDMJ), rats administered HK-MJ2 (10^8^ cells/ml) orally and DEX (2 mg/kg) intraperitoneally; Group 6: low-dose live-MJ2 (LDLMJ), rats administered live-MJ2 (10^7^ CFU/ml) orally and DEX (2 mg/kg) intraperitoneally; Group 7: high-dose live-MJ2 (HDLMJ), rats administered live-MJ2 (10^8^ CFU/ml) orally and DEX (2 mg/kg) intraperitoneally. LEU is known to improve muscle atrophy; thus, it was used as the test control [18, 19]. To induce muscle atrophy, rats in all groups, except for the NC group, were intraperitoneally injected with 2 mg/kg DEX for 7 days. Subsequently, the rats in all groups were administered their respective test materials orally and (2 mg/kg) intraperitoneally. Body weight measurements were conducted every 7 days. Before euthanizing the rats, their body weight was recorded, after which they were humanely euthanized. The soleus and gastrocnemius muscles from the hind leg were collected, weighed, immediately frozen in liquid nitrogen, and stored at -80°C until further use.

### Measurement of Grip Strength

Grip strength was measured using a grip strength meter (DBL Co., Republic of Korea), according to the manufacturer’s instructions. The rat tail was positioned to hold the grip length meter bar with its forelegs and then smoothly pulled back until the rat released its grip on the grid. The grip strength test was conducted thrice for each rat, on days 7, 14, and 21 after the administration of the test samples and immediately before sacrifice.

### Serum Biochemistry

After a 12-h fasting period, the rats were euthanized through cardiac puncture under isoflurane (2%) gas anesthesia. Blood samples were obtained and centrifuged at 3,000 ×g for 10 min to separate the serum. Liver toxicity was evaluated by measuring the levels of aspartate aminotransferase (AST) and alanine aminotransferase (ALT). Additionally, two muscle atrophy indicators, creatinine (CRE) and blood urea nitrogen (BUN), were analyzed using a FUJI DRI-CHEM 4000i (FUJIFILM Co., Japan).

### Measurement of Muscle Fiber Cross-Sectional Area

Soleus muscles were fixed in 4% formaldehyde and subsequently embedded in paraffin. The paraffin-embedded tissues were sliced into 5-μm-thick sections and subjected to hematoxylin and eosin staining. The cross-sectional area of the muscle fibers was determined using a light microscope (Leica DM750) (Leica Microsystems GmbH, Germany) by randomly measuring 50 cross-sectional areas for each sample and calculating the resultant average.

### Statistical Analysis

Statistical analyses were performed using the IBM SPSS Statistics version 25 (SPSS Inc. software (USA). All experiments were independently conducted at least three times. In vitro and *in vivo* experimental data are represented as the mean ± standard deviation (SD) and the mean ± standard error of mean (SEM), respectively. Differences among groups were determined using one-way analysis of variance (ANOVA), followed by Tukey’s honestly significant difference (HSD) post-hoc test. Differences were considered statistically significant at *p* < 0.05.

## Results and Discussion

### Cytotoxicity of HK-MJ2 against C2C12 Cells

**Fig. 1:**
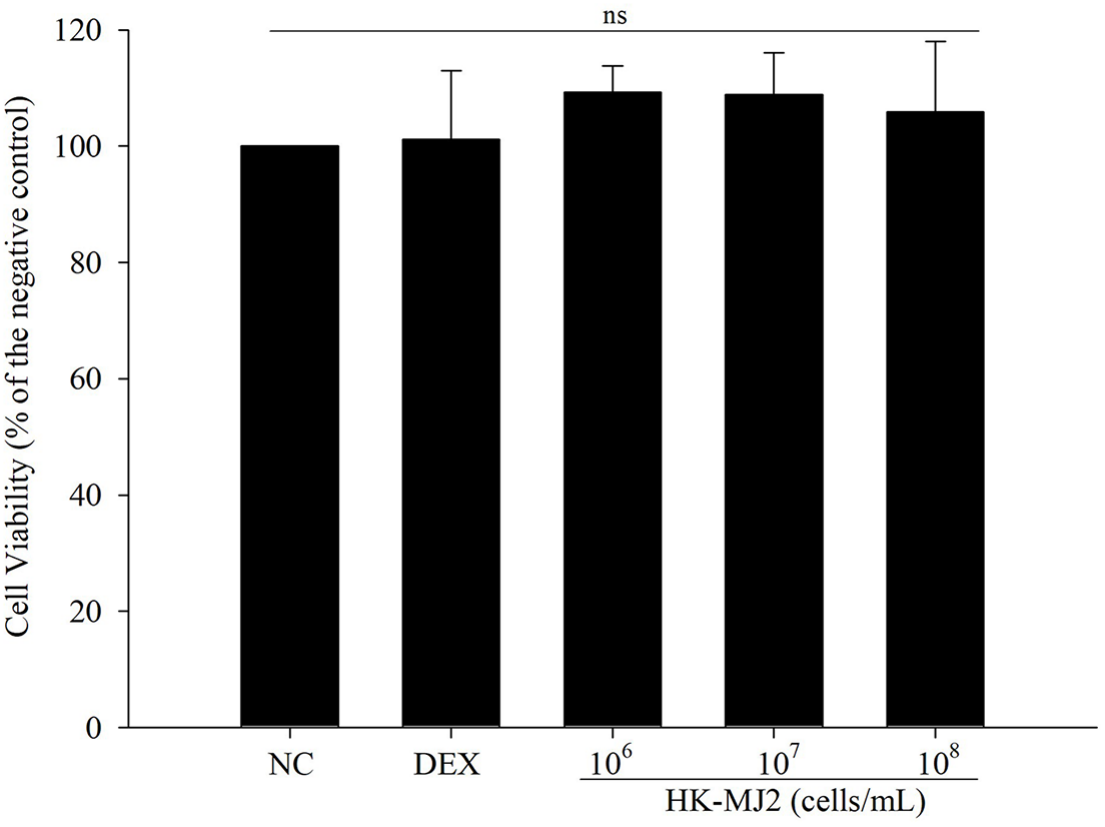
Cell viability of differentiated C2C12 cells treated with DEX and various concentrations of HK-MJ2. Cell viability was measured by MTT assay. The data indicate the mean ± SD of three independent experiments performed in triplicate. One-way ANOVA/Tukey HSD analysis was performed. ns, not significant. NC, negative control; DEX, dexamethasone (100 μM)-only-treated.

Glucocorticoid DEX is a drug primarily utilized to inhibit inflammation and address brain disorders caused by glucocorticoid receptor dysregulation [20, 21]. However, high-dose DEX treatment increases muscle protein degradation and reduces muscle protein synthesis, leading to muscle atrophy [22]. Therefore, high-dose DEX is commonly employed to establish *in vitro* and *in vivo* models of muscle atrophy. In this study, the treatment with 100 μM DEX did not cause significant cytotoxicity compared to the negative control group (NC), indicating that this concentration did not cause cell death. In our preliminary experiment, we confirmed that DEX (100 mM) induced muscle atrophy. Therefore, in this study, we used a high dose of DEX (100 μM) for subsequent *in vitro* experiments to induce muscle atrophy without causing cell death. Furthermore, the viability of differentiated C2C12 cells treated with HK-MJ2 and DEX was not significantly different from that of NC (Fig. 1). Thus, for *in vitro* experiments in this study, HK-MJ2 was employed at concentrations of 10^6^, 10^7^, and 10^8^ cells/ml.

### HK-MJ2 Inhibits DEX-Induced Reduction of Myotube Diameter in Differentiated C2C12 Myotubes

**Fig. 2:**
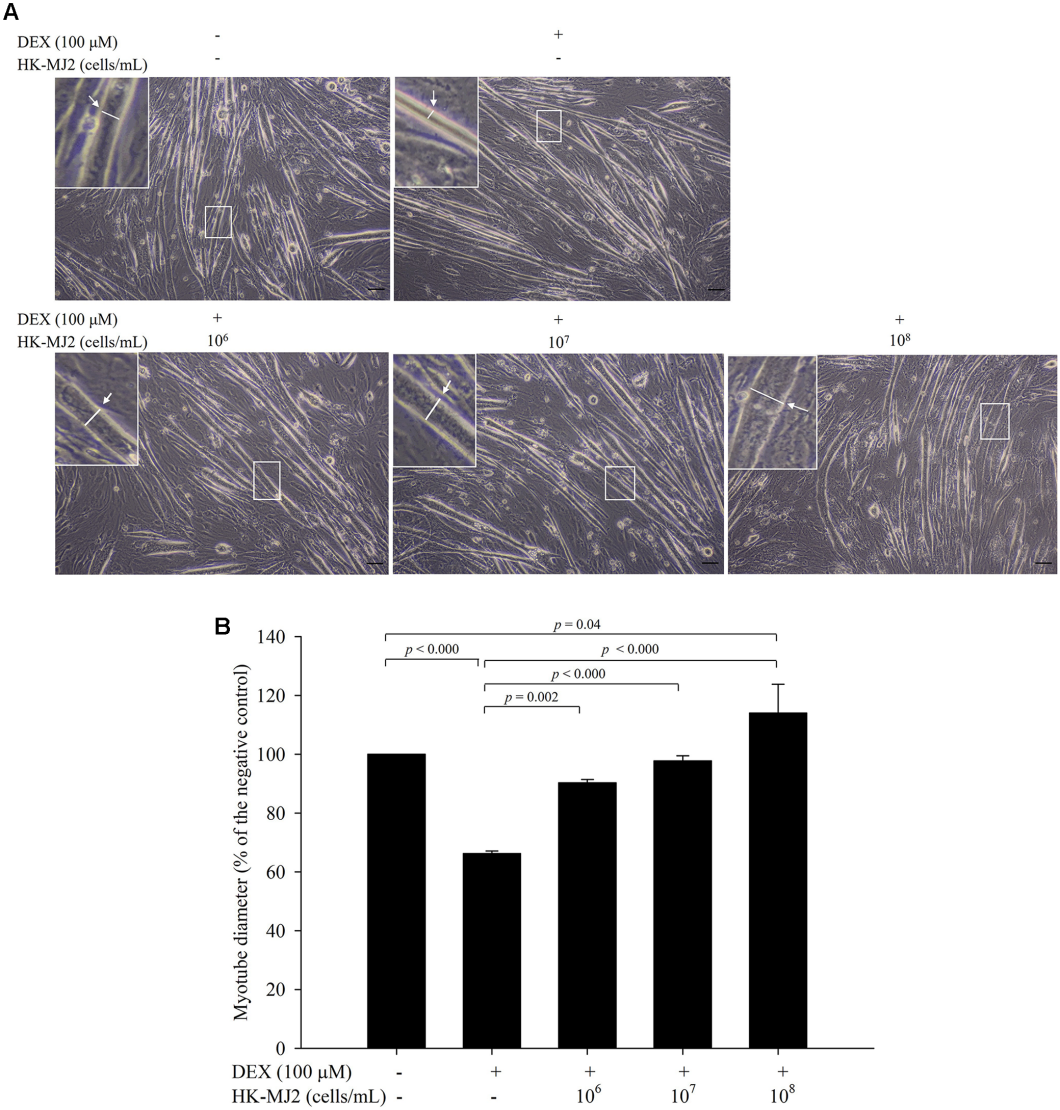
Inhibitory effect of HK-MJ2 on the reduction of myotube diameter in DEX-treated C2C12 myotubes. (**A**) Differentiation cell myotubes were visualized through microscopy (magnification 100×, Scale bar = 100 μm). Boxed area is enlarged in the upper left corner and the representative myotubes and their diameters are indicated with white arrows and lines. (**B**) Quantification of myotube diameter in differentiated cells was performed using the ImageJ software program (n = 50 myotubes). The presented data indicate the mean ± SD of three independent experiments. The p values were calculated using one-way analysis of variance (ANOVA)/Tukey’s honestly significant difference (HSD) analysis.

Adult skeletal muscle regeneration is accompanied by the formation of new myotubes. The myotube diameter serves as an indicator of recovery from skeletal muscle damage. In certain conditions, such as sarcopenia and chronic obstructive pulmonary disease (COPD), the myotube diameter reduces, particularly in aged muscle cells that are unable to restore the reduced myotube diameter [23, 24]. To assess the anti-atrophic effect of HK-MJ2 on DEX-treated myotubes, myotube diameter measurements were conducted. Notably, myotube diameter significantly reduced by 33.7 ± 0.9% when treated with DEX alone, compared to the negative control (Fig. 2). In contrast, in the HK-MJ2-treated groups with, the DEX-induced decrease in myotube diameter showed significant dose-dependent inhibition, compared to the group treated solely with DEX. Furthermore, the group treated with 10^8^ cells/ml HK-MJ2 exhibited a significantly higher cell count compared to the negative control. These results suggest that HK-MJ2 has the potential to alleviate muscle atrophy.

### Effects of HK-MJ2 on Skeletal Muscle Protein Regulation in DEX-Treated Differentiated C2C12 Cells

**Fig. 3:**
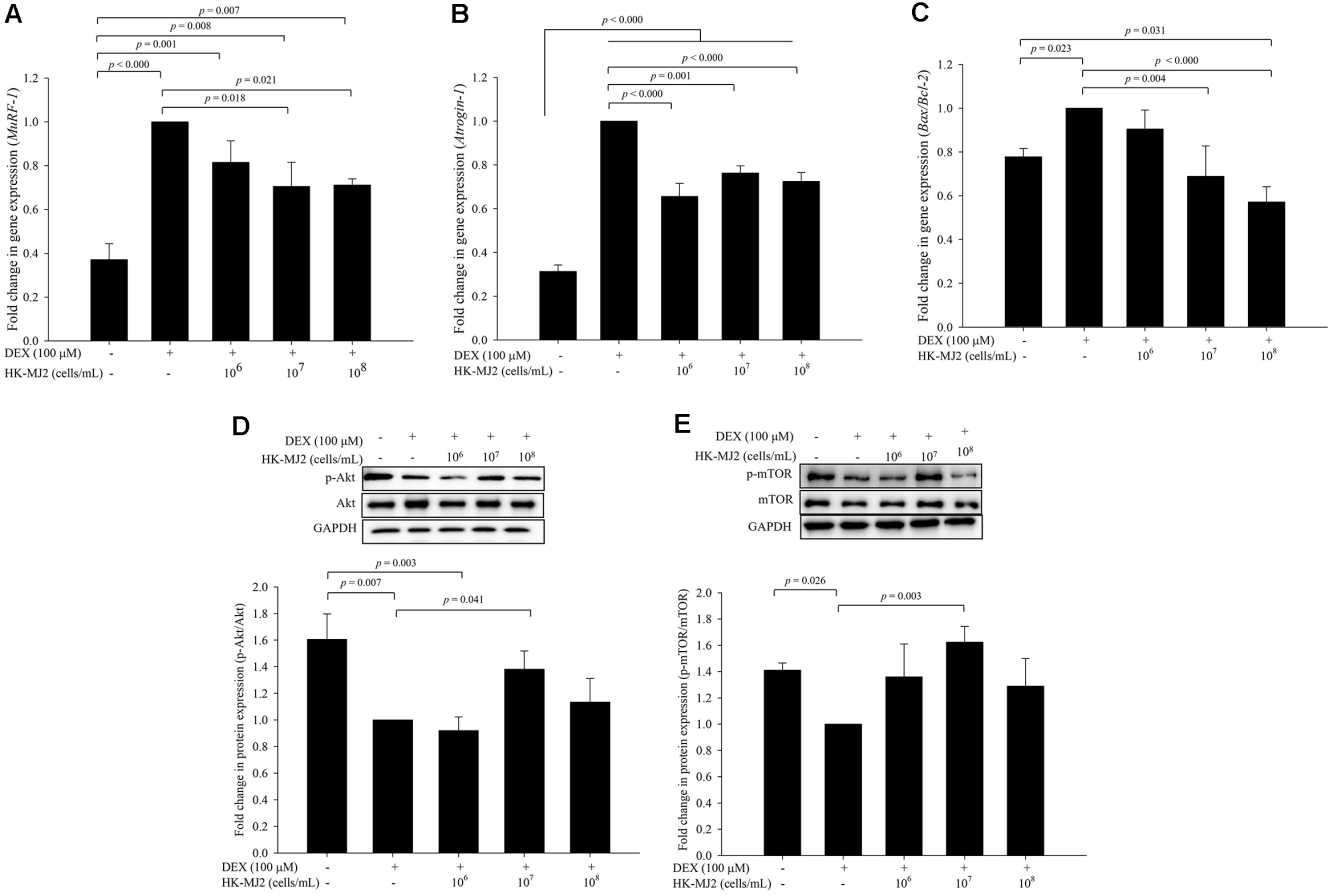
Effects of HK-MJ2 on the expression levels of muscle protein degradation genes and muscle protein synthesis factors in DEX-treated C2C12 myotubes. The mRNA expression levels of factors related to muscle protein degradation (**A**, *MuRF-1*; **B**, *Atrogin-1*; **C**, *Bax/Bcl-2*) in differentiated C2C12 cells treated with DEX and HK-MJ2 for 24 h were measured by quantitative real-time polymerase chain reaction (qPCR). Furthermore, the expression levels of p- Akt/Akt (**D**) and p-mTOR/mTOR (**E**) were evaluated by western blotting and quantified. The presented data indicate the mean ± SD of three independent experiments in triplicate. The p values were calculated using one-way analysis of variance (ANOVA)/Tukey’s honestly significant difference (HSD) analysis.

Muscle atrophy is caused by the activation of the ubiquitin–proteasome system, which is the core of skeletal muscle protein regulation. Specifically, an increase in FOXO3a and E3 ubiquitin ligases (MuRF-1 and Atrogin-1) promotes muscle protein degradation and accelerates muscle atrophy [25]. DEX induces muscle atrophy through its involvement in the ubiquitin-proteasome system. MuRF-1 and Atrogin-1 are important factors in ubiquitin-mediated muscle proteolysis, while Bax/Bcl-2 is a biomarker of apoptosis. To evaluate the inhibitory effect of HK-MJ2 on muscle proteolysis and muscle cell apoptosis, the expression levels of genes related to muscle protein degradation in differentiated C2C12 cells were measured using qPCR. The expression level of *MuRF-1* significantly increased 2.7-fold in the DEX-only-treated cells compared to the negative control. However, this increase was significantly reduced in the cells treated with HK-MJ2, except for the 10^6^ cells/ml HK-MJ2-treated cells (Fig. 3A). The expression level of *Atrogin-1*, which increased 3.2-fold in the DEX-only-treated cells compared to the negative control, significantly decreased in all HK-MJ2-treated cells compared to the DEX-only-treated group (Fig. 3B). Similarly, the expression level of *Bax/Bcl-2* increased 1.3-fold in the DEX-only-treated cells compared to the negative control, yet significantly decreased in the cells treated with HK-MJ2, except for the 10^6^ cells/ml HK-MJ2-treated cells (Fig. 3C). In addition, the expression level of *Bax/Bcl-2* in the 10^8^ cells/mL HK-MJ2-treated cells significantly decreased by 0.73-fold compared to the negative control. Therefore, HK-MJ2 effectively inhibited the DEX-induced upregulation of the expression of *Atrogin-1* and *MuRF-1*, which are the main factors responsible for protein degradation-driven muscle atrophy induced in skeletal muscle, thus preventing muscle cell death that can cause muscle atrophy.

Furthermore, the activation of both proteins (Akt and mTOR) in the DEX-treated cells significantly decreased compared to the negative control cells; however, this decreased expression appeared to be alleviated in the HK-MJ2-treated groups (Figs. 3D and 2E). Notably, the activation of both proteins showed a significant increase in the 10^7^ cells/ml HK-MJ2-treated cells compared to that in the DEX-only-treated group. Although no significant activation was observed in the 10^6^ and 10^8^ cells/ml HK-MJ2-treated groups, treatment with HK-MJ2 showed a tendency to recover the DEX-induced reduction in Akt and mTOR activation. Given that the activation of Akt and mTOR proteins plays a pivotal role in signal transduction affecting muscle protein synthesis [26], it is plausible that HK-MJ2 may induce muscle protein synthesis by activating the Akt/mTOR signaling pathway.

### Effects of live- and HK-MJ2 on Body Weight, Muscle Mass, and Cross-Sectional Area of Muscle Fiber in Rats with Induced Muscle Atrophy

To validate the effect of live- and HK-MJ2 on alleviating DEX-induced muscle atrophy, body weight and muscle mass were measured in rats that developed muscle atrophy subsequent to intraperitoneal DEX injection. The administration of DEX led to a noticeable reduction in body weight; however, both live- and HK-MJ2 treatments exhibited a progressive recovery of body weight across all MJ2-treated groups. After 28 days, the body weights in all MJ2-treated groups and the LEU group showed no significant reduction compared to the NC group (Table 1). In contrast, the body weight of the DEX group significantly decreased when compared with the NC group.

**Fig. 4:**
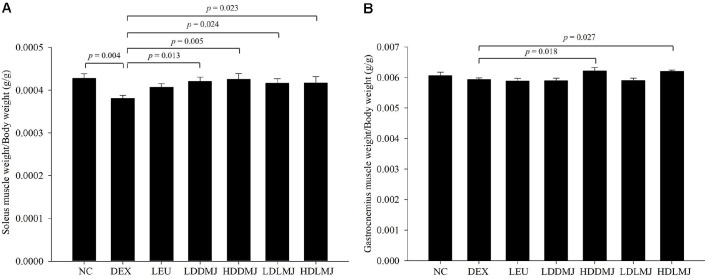
Inhibitory effects of live- and HK-MJ2 on muscle mass reduction in rats with DEX-induced muscle atrophy. The soleus (**A**) and gastrocnemius (**B**) muscles were carefully excised and weighed. The presented data indicate the mean ± SEM. The p values were calculated using one-way analysis of variance (ANOVA)/Tukey’s honestly significant difference (HSD) analysis. NC, normal control; DEX, dexamethasone (2 mg/kg)-only-treated; LEU, test control, DEX + leucine (600 mg/kg); LDDMJ, DEX + low-dose HK-MJ2 (10^7^ cells/ml); HDDMJ, DEX + high-dose HK-MJ2 (10^8^ cells/ml); LDLMJ, DEX + low-dose live-MJ2 (10^7^ CFU/ml); HDLMJ, DEX + high-dose live-MJ2 (10^8^ CFU/ml).

DEX accelerates the degradation of muscle proteins, leading to muscle atrophy by causing a reduction in muscle mass, muscle fiber cross-sectional area, and muscle strength [27]. The soleus and gastrocnemius muscles, which are composite-fiber muscles including both slow-twitch type I and fast-twitch type II fibers, are used as indicators to evaluate muscle atrophy [28, 29]. Specifically, the soleus muscle weight exhibited a significant reduction in the DEX-only-treated group compared to the NC group (Fig. 4A). However, in all MJ2-treated groups, the soleus muscle weights significantly increased compared to the DEX-only-treated group. In the gastrocnemius muscle, although no significant difference was observed between the DEX-only-treated and NC groups, discernible weight increments were evident in the high-dose MJ2-treated groups (HDDMJ and HDLMJ) when compared to the DEX-only-treated group (Fig. 4B). Consequently, MJ2 effectively inhibits the loss of body weight and muscle caused by DEX-induced muscle atrophy in rats. The Akt and mTOR signaling pathways, which affect muscle protein synthesis, respond to fast-twitch type II and slow-twitch type I fibers. Type I fibers are particularly susceptible to atrophy [26], with the soleus muscle being predominantly composed of type 1 slow muscle fibers [30]. In this study, we observed that the soleus muscle experienced more pronounced atrophy in response to DEX when compared to the gastrocnemius muscle. Moreover, MJ2 showed a notably stronger anti-atrophic effect on the soleus muscle as opposed in the gastrocnemius muscle.

**Fig. 5:**
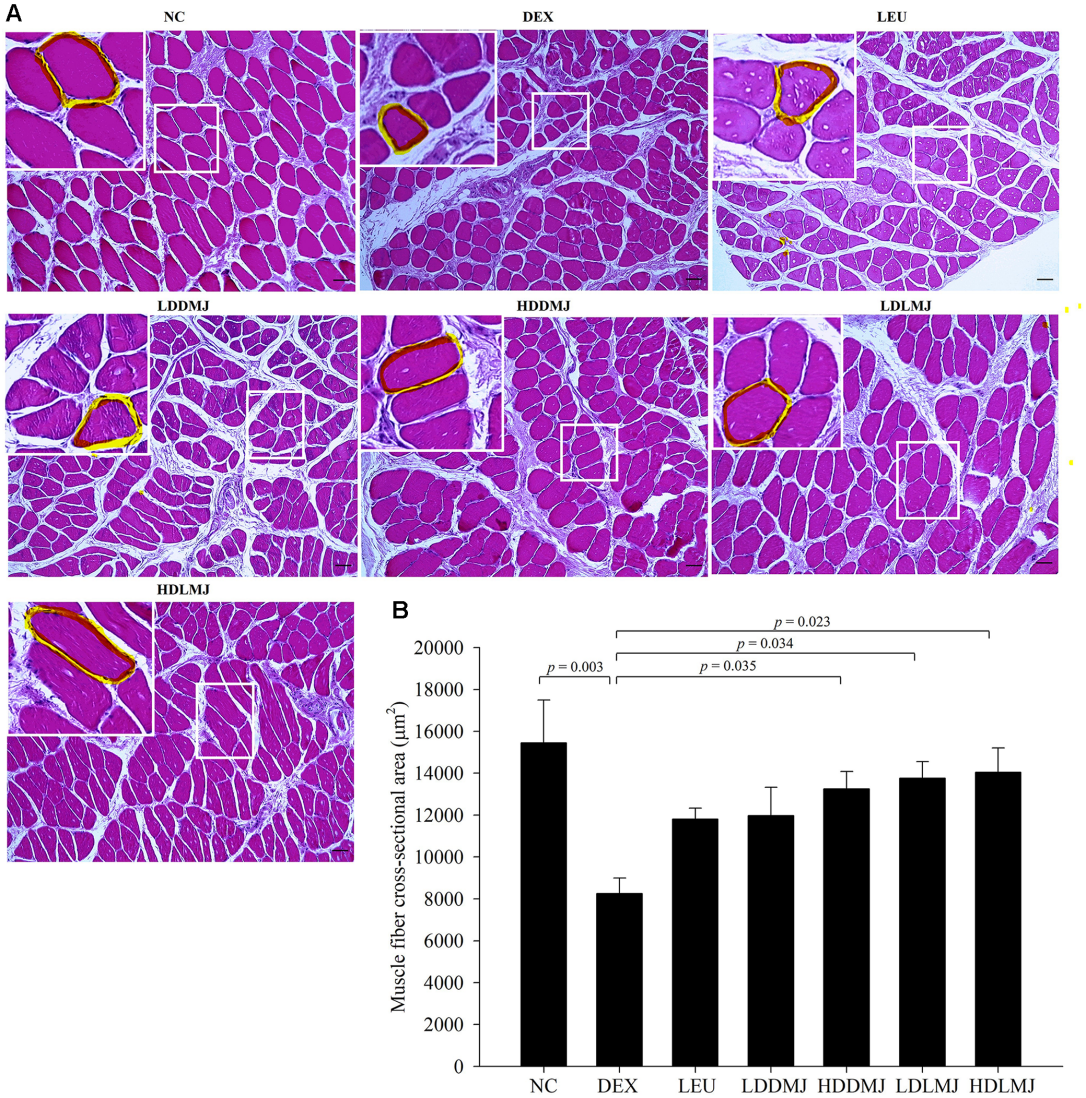
Inhibitory Effects of live- and HK-MJ2 on muscle fiber size reduction in rats with DEX-induced muscle atrophy. (**A**) Histological sections of excised soleus muscle tissue stained with H&E (magnification 100×, Scale bar = 100 μm). Boxed area is the representative cross-sectional area and is enlarged in the upper left corner. Each representative cross-sectional area is marked by a yellow line. (**B**) Quantitative analysis of the cross-sectional area of soleus muscle fibers. The presented data indicate the mean ± SEM. The p values were calculated using one-way analysis of variance (ANOVA)/Tukey’s honestly significant difference (HSD) analysis. NC, normal control; DEX, dexamethasone (2 mg/kg) only-treated; LEU, test control, DEX + leucine (600 mg/kg); LDDMJ, DEX + low-dose HKMJ2 (10^7^ cells/ml); HDDMJ, DEX + high-dose HK-MJ2 (10^8^ cells/ml); LDLMJ, DEX + low-dose live-MJ2 (10^7^ CFU/ml); HDLMJ, DEX + high-dose live-MJ2 (10^8^ CFU/ml).

To evaluate the inhibitory effect of MJ2 on muscle atrophy, we employed H&E staining to measure muscle fiber cross-sectional areas (Fig. 5A). Notably, the cross-sectional area of the DEX-only-treated group significantly decreased by 46.6 ± 11.0% in comparison to the NC group. Conversely, in all MJ2-treated groups, except for the LDDMJ group and LEU groups, a significant increase in cross-sectional area was observed when compared with the DEX-only-treated group (Fig. 5B). These findings show that both live- and HK-MJ2 treatments effectively prevent the reduction in muscle cell size, suggesting that MJ2 treatment holds promise for ameliorating DEX-induced muscle atrophy.

Branched-chain amino acids have been shown to mitigate DEX-induced muscle atrophy, as assessed through evaluations of the soleus and gastrocnemius muscles [31]. Among these, leucine, recognized for its protein anabolic properties, has shown anti-atrophic effects, particularly at high doses [18, 19]. In this study, MJ2 effectively prevented and improved muscle atrophy by inhibiting the decrease in muscle weight and muscle fiber cross-sectional area and was more effective in improving muscle atrophy than leucine.

### Effect of MJ2 on Grip Strength and Blood Chemical Factors in Rats with Induced Muscle Atrophy

DEX reduces grip strength in rats via muscle atrophy, making grip strength a valid indicator of muscle atrophy [6]. Grip strength measurements were conducted to evaluate the inhibitory effects of MJ2 on DEX-induced muscle atrophy in rats. After 14 days of DEX treatment, grip strength decreased across all groups except for the NC group (Table 2). However, after 28 days of treatment with live- or HK-MJ2 with DEX, although the grip strengths in all MJ2-treated groups, except for the HDDMJ group, remained significantly lower than those of the NC group, a significant increase in grip strengths was observed across all MJ2-treated groups compared to the DEX-only-treated group. These findings showed that MJ2 treatment increased the decrease in muscle strength caused by DEX-induced muscle atrophy.

Serum creatinine (CRE) and blood urea nitrogen (BUN) levels, which increase with muscle damage and degradation, respectively, serve as serum biomarkers for assessing skeletal muscle atrophy. Elevated serum CRE and BUN levels indicative of muscle atrophy in animal models [32, 33]. Specifically, the serum levels of CRE and BUN were significantly higher in the DEX-only-treated group than in the NC group (Table 2). Notably, while CRE levels in all MJ2-treated groups, except for the LDDMJ group, exhibited a significant decrease compared to the DEX-only-treated group, the MJ2-treated groups showed no significant difference from the NC group. Serum BUN levels of all MJ2-treated groups and the LEU group were significantly decreased compared to the DEX group and showed no significant difference from the NC group. These findings suggest that the increased serum CRE and BUN levels, indicative of DEX-induced muscle protein degradation, were significantly reduced by live- or HK-MJ2 treatment. This suggests that both live- and HK-MJ2 treatments effectively inhibit muscle damage and degradation, ultimately leading to the amelioration of DEX-induced muscle atrophy.

AST and ALT levels were measured to evaluate the liver toxicity of MJ2 treatment. No significant difference in AST and ALT levels was observed across all groups (Table 3). These results indicate that live- and HK-MJ2 treatment did not induce hepatotoxicity in rats with DEX-induced muscle atrophy.

The expression of E3 ubiquitin ligases (MuRF-1 and Atrogin-1), which play a major role in DEX-induced muscle atrophy through ubiquitin-mediated protein degradation. These ligases serve as important regulators of ubiquitin–proteasome system activation in muscle cells. In this study, we confirmed that DEX treatment increased the expression levels of MuRF-1 and Atrogin-1 in C2C12 cells and MJ2 treatment downregulated E3 ubiquitin ligases. These findings strongly indicate that MJ2 treatment effectively inhibits protein degradation and prevents muscle atrophy induction, thereby inhibiting the development of muscle atrophy. Furthermore, the AKT/mTOR pathway is closely linked to muscle protein synthesis [34, 35]. DEX treatment hampers the Akt/mTOR signaling pathways associated with protein synthesis, resulting in increased muscle protein degradation and a reduced muscle protein synthesis, thus causing muscle atrophy [25]. In this study, the application of HK-MJ2 treatment, especially at a concentration of 10^7^ cells/ml, restored the activation of Akt and mTOR, which was blocked by DEX treatment. The concentration of 10^7^ cells/ml HK-MJ2 may represent the optimal window for activating the specific Akt/mTOR phosphorylation cascade in this *in vitro* model. The higher concentration (10^8^ cells/ml HK-MJ2) may induce feedback inhibition loops or C2C12 cell surface receptor saturation, leading to lower activity than 10^7^ cells/ml HK-MJ2-treated cells. However, structurally, the treatment of 10^8^ cells/ml HK-MJ2 still effectively maintained myotube diameter, implying that muscle mass preservation may involve composite mechanisms beyond just the peak activation of Akt/mTOR observed at a single time point (10^7^ cells/ml HK-MJ2) in this study. Despite being metabolically inactivated, HK-MJ2 might retain heat-stable cell wall components, such as peptidoglycans, teichoic acids, and some heat-stable surface proteins. These components could possibly interact with host pattern recognition receptors (*e.g.*, Toll-like receptors) on the C2C12 cell surface, consequently potentially modulating immune responses and activating intracellular signaling pathways like Akt/mTOR, which might contribute to atrophy-inhibitory effects of HK-MJ2.

Although we could not confirm this activation in the *in vivo* model, MJ2 treatment might have stimulated muscle protein synthesis, potentially leading to an increase in grip strength and muscle weight. Therefore, live- and HK-MJ2 improves muscle atrophy by regulating the degradation of muscle proteins through the downregulation of the DEX-activated ubiquitin–proteasome system.

**Fig. 6:**
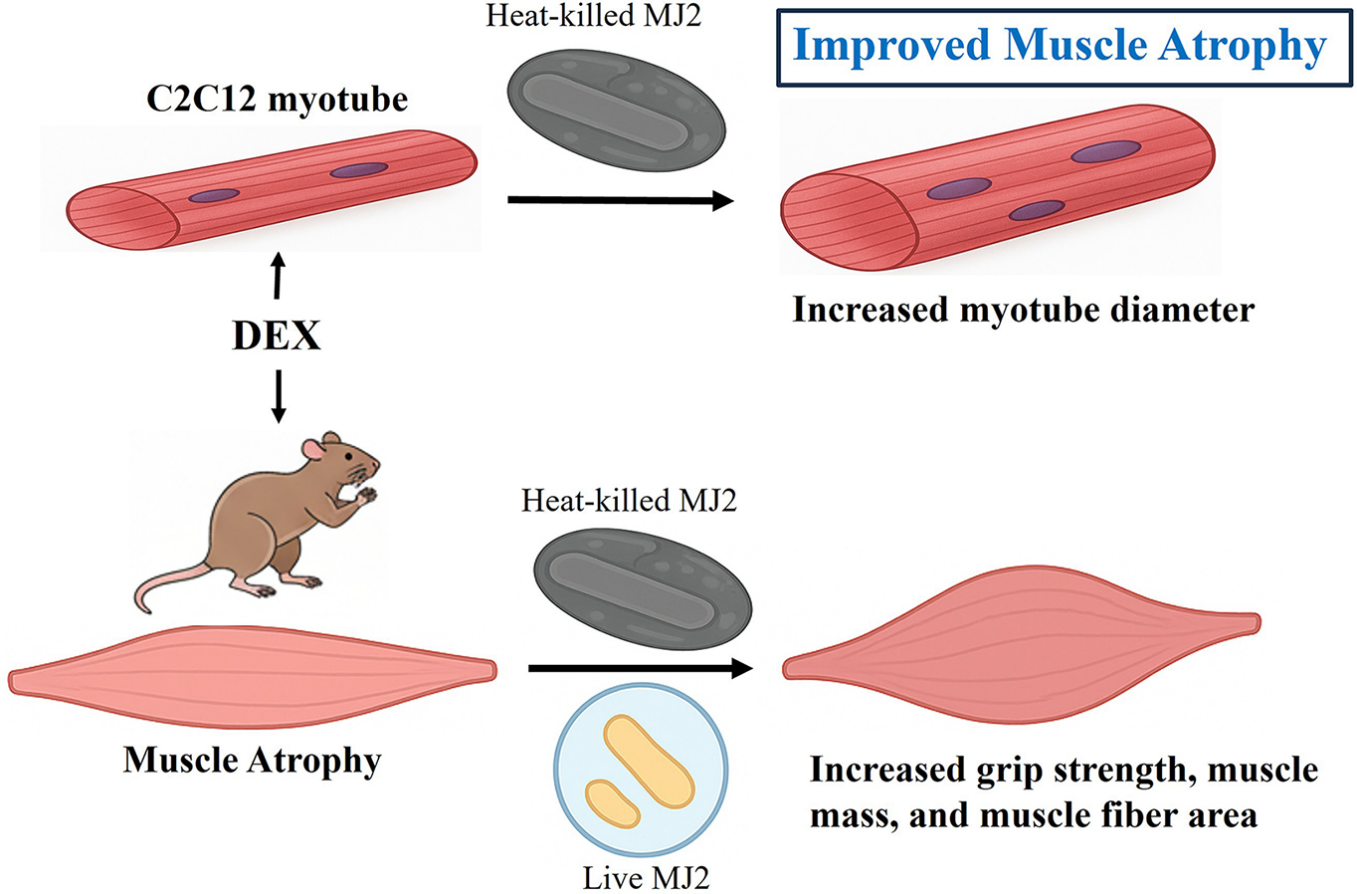
Schematic overview of the improvement effects of live- and HK-MJ2 on DEX-induced muscle atrophy in rats. HK-MJ2 increased myotube diameter of DEX-treated C2C12 myotubes. Both live- and HK-MJ2 increased grip strength, muscle mass, and muscle fiber area in rats with DEX-induced muscle atrophy.

Muscle loss and atrophy are closely correlated, and muscle atrophy caused by spinal diseases, cancer, and aging poses a significant challenge in the context of aging population [36, 37]. However, no effective drugs for treating muscle atrophy have been approved thus far. Although efforts are being made in the realm of natural compounds, such as resveratrol extract, salidroside, and matrine to address muscle atrophy, their effects remain insufficient. While certain drugs, such as thalidomide and myostatin inhibitors, have shown effectiveness in improving muscle atrophy, their practical utility is hampered by potential side effects [38]. In this study, the probiotic MJ2 isolated from raw milk showed a preventive effect on DEX-induced muscle atrophy. This might be achieved by suppressing the activation of the ubiquitin–proteasome system through the inhibition of the genes related to muscle protein degradation. Furthermore, MJ2 treatment ameliorated the decrease in grip strength and muscle weight caused by DEX-induced muscle atrophy, indicating that MJ2 effectively improved DEX-induced muscle atrophy. Based on the results obtained this study, we summarized the improved effect of MJ2 on DEX-induced muscle atrophy in rats (Fig. 6). HK-MJ2 inhibited the decrease of myotube diameter in DEX-treated C2C12 myotubes. Both live- and HK-MJ2 increased grip strength, muscle mass, and muscle fiber area resulted in the improvement of DEX-induced muscle atrophy in rats.

In conclusion, the probiotic strain MJ2, isolated from raw milk, holds promise for ameliorating muscle atrophy by increasing muscle mass and muscle fiber area. Interestingly, although HK-MJ2 showed greater efficacy in preventing muscle atrophy in rats with DEX-induced muscle atrophy, both live- and HK-MJ2 showed notable improvement effects on DEX-induced muscle atrophy. Notably, HK-MJ2 exhibited anti-atrophic effects comparable to live-MJ2, which suggests that HK-MJ2 can be served as an effective paraprobiotic. Since paraprobiotics eliminate stability concerns associated with live bacteria, HK-MJ2 can be a promising safe alternative for preventing muscle atrophy.

In this study, we primarily focused on the inhibition of catabolic pathways (ubiquitin-proteasome system) and apoptosis. Beside atrophy-related factors and protein synthesis signaling factors, activation of muscle regeneration markers such as MyoD and myogenin (MyoG) are important for muscle growth and regeneration. However, the expression of myogenic regulatory factors was not evaluated in this study, which is a limitation of our study. Further investigations, including myogenic regulatory factors and transcriptomic profiling, are imperative to elucidate the mechanism *in vivo* and identify specific active substance(s) derived from MJ2 that are responsible for its anti-atrophic effects.

## Figures and Tables

**Table 1:** Live- and HK-MJ2 inhibited body weight reduction in the muscle atrophy-induced rats.

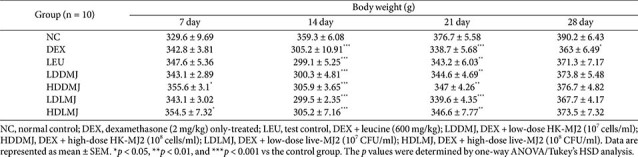

**Table 2:** Effects of live- and HK-MJ2 on grip strength and blood chemical factors in DEX-induced muscle atrophy in rats.

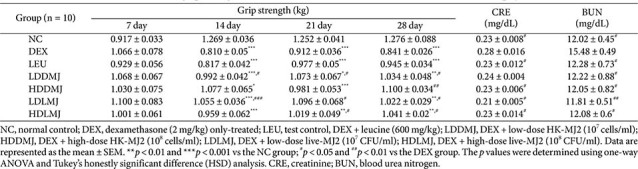

**Table 3:** Hepatotoxicity of live- and HK-MJ2 in DEX-induced muscle atrophy in rats.

Group (n = 6)	AST (U/L)	ALT (U/L)
NC	109.83 ± 7.03^ns^	38.17 ± 3.74^ns^
DEX	75.00 ± 10.7	36.17 ± 5.22
LEU	80.00 ± 13.88	33.67 ± 6.51
LDDMJ	68.67 ± 6.79	33.00 ± 3.43
HDDMJ	75.33 ± 10.35	41.33 ± 6.06
LDLMJ	72.17 ± 9.35	36.33 ± 6.74
HDLMJ	70.50 ± 7.6	30.17 ± 4.02

Data are represented as mean ± SEM. One-way ANOVA/Tukey HSD analysis was performed. ns, not significant. AST, aspartate aminotransferase; ALT, alanine aminotransferase; NC, normal control; DEX, dexamethasone (2 mg/kg) only-treated; LEU, test control, DEX + leucine (600 mg/kg); LDDMJ, DEX + low-dose HKMJ2 (10^7^ cells/ml); HDDMJ, DEX + high-dose HK-MJ2 (10^8^ cells/ml); LDLMJ, DEX + low-dose live-MJ2 (10^7^ CFU/ml); HDLMJ, DEX + high-dose live-MJ2 (10^8^ CFU/ml).

## References

[1] Che J, Xu C, Wu Y, Jia P, Han Q, Ma Y (2021). MiR-1290 promotes myoblast differentiation and protects against myotube atrophy via Akt/p70/FoxO3 pathway regulation. Skelet. Muscle.

[2] Malavaki CJ, Sakkas GK, Mitrou GI, Kalyva A, Stefanidis I, Myburgh KH (2015). Skeletal muscle atrophy: disease-induced mechanisms may mask disuse atrophy. J. Muscle Res. Cell Motil..

[3] Rom O, Reznick AZ (2016). The role of E3 ubiquitin-ligases MuRF-1 and MAFbx in loss of skeletal muscle mass. Free Radic. Biol. Med..

[4] Kim S, Kim K, Park J, Jun W (2021). *Curcuma longa* L. water extract improves dexamethasone-induced sarcopenia by modulating the muscle-related gene and oxidative stress in mice. Antioxidants.

[5] Krug AL, Macedo AG, Zago AS, Rush JW, Santos CF, Amaral SL (2016). High-intensity resistance training attenuates dexamethasone-induced muscle atrophy. Muscle Nerve.

[6] Yeon M, Choi H, Jun HS (2020). Preventive effects of schisandrin A, a bioactive component of schisandra chinensis, on dexamethasone-induced muscle atrophy. Nutrients.

[7] Cohen S, Nathan JA, Goldberg AL (2015). Muscle wasting in disease: molecular mechanisms and promising therapies. Nat. Rev. Drug Discov..

[8] Terracciano C, Celi M, Lecce D, Baldi J, Rastelli E, Lena E (2013). Differential features of muscle fiber atrophy in osteoporosis and osteoarthritis. Osteoporos. Int..

[9] Bettis T, Kim BJ, Hamrick MW (2018). Impact of muscle atrophy on bone metabolism and bone strength: implications for muscle-bone crosstalk with aging and disuse. Osteoporos. Int..

[10] Prokopidis K, Giannos P, Kirwan R, Ispoglou T, Galli F, Witard OC (2023). Impact of probiotics on muscle mass, muscle strength and lean mass: a systematic review and meta-analysis of randomized controlled trials. J. Cachexia Sarcopenia Muscle.

[11] Giron M, Thomas M, Dardevet D, Chassard C, Savary-Auzeloux I (2022). Gut microbes and muscle function: can probiotics make our muscles stronger. J. Cachexia Sarcopenia Muscle.

[12] Adams CA (2010). The probiotic paradox: live and dead cells are biological response modifiers. Nutr. Res. Rev..

[13] Lee CC, Liao YC, Lee MC, Cheng YC, Chiou SY, Lin JS (2022). Different impacts of heat-killed and viable *Lactiplantibacillus plantarum* TWK10 on exercise performance, fatigue, body composition, and gut microbiota in humans. Microorganisms.

[14] Poaty Ditengou JIC, Ahn SI, Chae B, Choi NJ (2023). Are heat-killed probiotics more effective than live ones on colon length shortness, disease activity index, and the histological score of an inflammatory bowel disease-induced murine model? A meta-analysis. J. Appl. Microbiol..

[15] An M, Park YH, Lim YH (2021). Antiobesity and antidiabetic effects of the dairy bacterium *Propionibacterium freudenreichii* MJ2 in high-fat diet-induced obese mice by modulating lipid metabolism. Sci. Rep..

[16] Yeom J, Ma S, Lim YH (2021). Probiotic *Propionibacterium freudenreichii* MJ2 enhances osteoblast differentiation and mineralization by increasing the OPG/RANKL ratio. Microorganisms.

[17] Livak KJ, Schmittgen TD (2001). Analysis of relative gene expression data using real-time quantitative PCR and the 2(-delta delta C(T)) method. Methods.

[18] Cruz A, Ferian A, Alves PKN, Silva WJ, Bento MR, Gasch A (2020). Skeletal muscle anti-atrophic effects of leucine involve myostatin inhibition. DNA Cell Biol..

[19] Pereira MG, Silva MT, da Cunha FM, Moriscot AS, Aoki MS, Miyabara EH (2015). Leucine supplementation improves regeneration of skeletal muscles from old rats. Exp. Gerontol..

[20] Williams S, Ghosh C (2020). Neurovascular glucocorticoid receptors and glucocorticoids: implications in health, neurological disorders and drug therapy. Drug Discov. Today.

[21] Jesinkey SR, Korrapati MC, Rasbach KA, Beeson CC, Schnellmann RG (2014). Atomoxetine prevents dexamethasone-induced skeletal muscle atrophy in mice. J. Pharmacol. Exp. Ther..

[22] Burt MG, Johannsson G, Umpleby AM, Chisholm DJ, Ho KK (2007). Impact of acute and chronic low-dose glucocorticoids on protein metabolism. J. Clin. Endocrinol. Metab..

[23] Mankhong S, Kim S, Moon S, Kwak H-B, Park D-H, Kang J-H (2020). Experimental models of sarcopenia: bridging molecular mechanism and therapeutic strategy. Cells.

[24] Pomiès P, Rodriguez J, Blaquière M, Sedraoui S, Gouzi F, Carnac G (2015). Reduced myotube diameter, atrophic signalling and elevated oxidative stress in cultured satellite cells from COPD patients. J. Cell. Mol. Med..

[25] Stitt TN, Drujan D, Clarke BA, Panaro F, Timofeyva Y, Kline WO (2004). The IGF-1/PI3K/Akt pathway prevents expression of muscle atrophy-induced ubiquitin ligases by inhibiting FOXO transcription factors. Mol. Cell.

[26] Gao H, Li YF (2018). Distinct signal transductions in fast- and slow- twitch muscles upon denervation. Physiol. Rep..

[27] Qin J, Du R, Yang YQ, Zhang HQ, Li Q, Liu L (2013). Dexamethasone-induced skeletal muscle atrophy was associated with upregulation of myostatin promoter activity. Res. Vet. Sci..

[28] Baek KW, Jung YK, Kim JS, Park JS, Hah YS, Kim SJ (2020). Rodent model of muscular atrophy for sarcopenia study. J. Bone Metab..

[29] Mizunoe Y, Kobayashi M, Saito H, Goto A, Migitaka R, Miura K (2021). Prolonged caloric restriction ameliorates age-related atrophy in slow and fast muscle fibers of rat soleus muscle. Exp. Gerontol..

[30] Heikkinen J, Lantto I, Flinkkila T, Ohtonen P, Niinimaki J, Siira P (2017). Soleus atrophy is common after the nonsurgical treatment of acute achilles tendon ruptures: a randomized clinical trial comparing surgical and nonsurgical functional treatments. Am. J. Sports Med..

[31] Yamamoto D, Maki T, Herningtyas EH, Ikeshita N, Shibahara H, Sugiyama Y (2010). Branched-chain amino acids protect against dexamethasone-induced soleus muscle atrophy in rats. Muscle Nerve.

[32] das Neves W, Alves CRR, de Souza Borges AP, de Castro G (2021). Serum Creatinine as a Potential Biomarker of Skeletal Muscle Atrophy in Non-small Cell Lung Cancer Patients. Front. Physiol..

[33] Gu L, Wang Z, Zhang Y, Zhu N, Li J, Yang M (2022). TLR13 contributes to skeletal muscle atrophy by increasing insulin resistance in chronic kidney disease. Cell Prolif..

[34] Yang W, Huang J, Wu H, Wang Y, Du Z, Ling Y (2020). Molecular mechanisms of cancer cachexia-induced muscle atrophy. Mol. Med. Rep..

[35] Ziaaldini MM, Marzetti E, Picca A, Murlasits Z (2017). Biochemical pathways of sarcopenia and their modulation by physical exercise: a narrative review. Front. Med..

[36] Sartori R, Romanello V, Sandri M (2021). Mechanisms of muscle atrophy and hypertrophy: implications in health and disease. Nat. Commun..

[37] Larsson L, Degens H, Li M, Salviati L, Lee YI, Thompson W (2019). Sarcopenia: aging-related loss of muscle mass and function. Physiol. Rev..

[38] Linlin C, Hong Z, Mengyi C, Quanjun Y, Cheng G. 2021. Drugs for the Treatment of Muscle Atrophy, pp. 83-104. *In* Cseri J. (ed.), *Background and Management of Muscular Atrophy*. IntechOpen, London, UK.

